# Inflammation in Dry Eye Syndrome: Identification and Targeting of Oxylipin-Mediated Mechanisms

**DOI:** 10.3390/biomedicines8090344

**Published:** 2020-09-11

**Authors:** Dmitry V. Chistyakov, Olga S. Gancharova, Viktoriia E. Baksheeva, Veronika V. Tiulina, Sergei V. Goriainov, Nadezhda V. Azbukina, Marina S. Tsarkova, Andrey A. Zamyatnin, Pavel P. Philippov, Marina G. Sergeeva, Ivan I. Senin, Evgeni Yu. Zernii

**Affiliations:** 1 Belozersky Institute of Physico-Chemical Biology, Lomonosov Moscow State University, 119992 Moscow, Russia; olgancharova@belozersky.msu.ru (O.S.G.); vbaksheeva@belozersky.msu.ru (V.E.B.); tyulina_nika@list.ru (V.V.T.); zamyat@belozersky.msu.ru (A.A.Z.J.); pf@belozersky.msu.ru (P.P.P.); mg.sergeeva@gmail.com (M.G.S.); senin@belozersky.msu.ru (I.I.S.); 2 Skryabin Moscow State Academy of Veterinary Medicine and Biotechnology, 109472 Moscow, Russia; marina.tsarkova@gmail.com; 3 Shared Research and Education Center of the Peoples’ Friendship University of Russia (RUDN University), 117198 Moscow, Russia; goryainovs@list.ru; 4 Faculty of Bioengineering and Bioinformatics, Moscow Lomonosov State University, 119234 Moscow, Russia; ridernadya@gmail.com; 5 Institute of Molecular Medicine, Sechenov First Moscow State Medical University, 119991 Moscow, Russia

**Keywords:** dry eye syndrome, inflammation, oxidative stress, corneal damage, tear lipidome, 5-lipoxigenase, leukotriene B4, prostaglandins, dimethyl sulfoxide, zileuton

## Abstract

Dry eye syndrome (DES) is characterized by decreased tear production and stability, leading to desiccating stress, inflammation and corneal damage. DES treatment may involve targeting the contributing inflammatory pathways mediated by polyunsaturated fatty acids and their derivatives, oxylipins. Here, using an animal model of general anesthesia-induced DES, we addressed these pathways by characterizing inflammatory changes in tear lipidome, in correlation with pathophysiological and biochemical signs of the disease. The decline in tear production was associated with the infiltration of inflammatory cells in the corneal stroma, which manifested one to three days after anesthesia, accompanied by changes in tear antioxidants and cytokines, resulting in persistent damage to the corneal epithelium. The inflammatory response manifested in the tear fluid as a short-term increase in linoleic and alpha-linolenic acid-derived oxylipins, followed by elevation in arachidonic acid and its derivatives, leukotriene B4 (5-lipoxigenase product), 12-hydroxyeicosatetraenoic acid (12-lipoxigeanse product) and prostaglandins, D2, E2 and F2α (cyclooxygenase products) that was observed for up to 7 days. Given these data, DES was treated by a novel ophthalmic formulation containing a dimethyl sulfoxide-based solution of zileuton, an inhibitor of 5-lipoxigenase and arachidonic acid release. The therapy markedly improved the corneal state in DES by attenuating cytokine- and oxylipin-mediated inflammatory responses, without affecting tear production rates. Interestingly, the high efficacy of the proposed therapy resulted from the synergetic action of its components, namely, the general healing activity of dimethyl sulfoxide, suppressing prostaglandins and the more specific effect of zileuton, downregulating leukotriene B4 (inhibition of T-cell recruitment), as well as upregulating docosahexaenoic acid (activation of resolution pathways).

## 1. Introduction

Dry eye syndrome (DES; also known as dry eye, dry eye disease, or keratoconjunctivitis sicca) is a common multifactorial ocular surface disease, characterized by decreased production/increased evaporation of the tear, resulting in its hyperosmolarity and instability of the tear film [1]. DES affects up to 40% of the adult population and manifests as eye irritation, hyperemia, glare, eye fatigue, and blurred vision [2,3]. Etiologically, alterations in tear homeostasis are associated with dysfunction of the lachrymal and/or Meibomian glands (such as in Sjögren syndrome and Meibomian Gland Dysfunction (MGD)) as well as a number of other factors, including contact lens wearing, adverse effects of various medications, complications of ocular surgery and general anesthesia [1,4,5,6,7].

In DES pathogenesis, the reduced tear production and/or alterations in the tear composition results in the loss of lubricating, nourishing and protective qualities of tears against the ocular surface tissues [8]. The most critical consequences of this desiccating stress are ocular surface inflammation and damage, which in severe cases can cause blindness, due to corneal scarring, opacification or ulceration [3]. Consistently, the generally accepted treatment of DES involves not only using lubricating eye drops and ointments, but also topical anti-inflammatory therapy [9,10]. Unfortunately, the administration of common ocular anti-inflammatory medications, such as corticosteroid eye drops or cyclosporine, is associated with a number of adverse effects in patients with dry eye, which generally limits their employment [11]. Thus, there is a great demand for a new safe and effective anti-inflammatory therapy for DES.

The solution to this problem requires an understanding of the mechanisms underlying inflammation in DES, which can be selectively targeted. Currently, it is recognized that an inflammatory reaction in DES is triggered by cytokines (such as interleukin (IL) 1 beta and tumor necrosis factor alpha (TNF-α)) secreted in tear fluid (TF) during the early stages of the disease. Indeed, hyperosmolarity of the tear film stimulates an inflammatory cascade, resulting in the release of these cytokines by limbal epithelial cells [12]. In addition, the desiccating stress leads to compensatory reflex stimulation of the lachrymal gland, which may activate a neurogenic inflammatory cytokine response [3,8]. These events induce maturation and an increase in the density of antigen-presenting dendritic cells in the cornea, subsequent activation of T-cells and their recruitment to the ocular surface (including conjunctiva and lacrimal glands) and release of additional effector cytokines in the TF, thereby causing further damage to the corneal epithelium [3,13]. These processes are accompanied by infiltration of the cornea by leucocytes and other immune cells [3,14]. Consistently, DES can be treated by using drugs blocking the activation of T cells (i.e., lifitegrast, LFA-1 antagonist) or inhibiting cytokine production by these cells (i.e., cyclosporine, suppressor of calcineurin regulated transcription of cytokine genes), as well as using cytokine receptor inhibitors [13,15,16]. Inflammatory changes in DES are also associated with oxidative stress of the ocular surface tissues, stemming from TF deficiency. Indeed, TF contains low molecular weight antioxidants (glutathione, ascorbic acid, and others) and reactive oxygen species (ROS) scavenging enzymes (glutathione reductase, glutathione peroxidase, superoxide dismutase, etc.) thereby providing local antioxidant defense [17,18]. Accordingly, the inflammatory component of DES can be attenuated by the topical administration of antioxidants [6,19].

The separate classes of molecules that contribute to inflammatory responses in DES and can be targeted in its prospective therapy are polyunsaturated fatty acids (PUFAs) and their derivative oxylipins, representing multipotent lipid mediators of inflammation. Oxylipins are biosynthesized from PUFAs via multiple oxidative reactions catalyzed by specific enzymes (cyclooxygenase (COX), lipoxygenase (LOX) or cytochrome P450 monooxygenase (CYP)) or proceeding in an enzyme-independent manner in the presence of ROS [20]. Previously, it was suggested that chronic inflammation in DES is associated with an imbalance between omega-3 (docosahexaenoic acid (DHA) and eicosapentaenoic acid (EPA)) and omega-6 (arachidonic acid (AA)) PUFAs, which lead to the hyperproduction of proinflammatory lipid mediators (omega-6 derivative oxylipins, such as prostaglandins) and underproduction of proresolving (omega-3 derivative oxylipis) molecules [21]. Consistently, DES patients were characterized by upregulation of prostaglandin (PG) E2 in TF, the levels of which correlated with their symptom grades [21,22,23]. Interestingly, selective inhibition of COX-2, responsible for PGE2 biosynthesis, reduced the levels of pro-inflammatory cytokines in DES apparently via the attenuating effect of PGE2 on antigen-presenting dendritic cells [24], indicating the existence of interrelation between inflammatory pathways in DES. Although COX suppression seems to be a promising route in DES therapy, the well-acknowledged inhibitors of cyclooxygenases, non-steroidal anti-inflammatory drugs (NSAIDs), are not recommended in patients with DES, due to a number of severe side effects [25,26]. Thus, it is important to develop an alternative approach to selective targeting of the oxylipin-dependent inflammatory pathways, based on an understanding of the dynamic changes of the full spectrum of these lipid mediators in DES.

Previously, we developed a rabbit model of DES, closely reproducing the pathogenesis of the human disease [27]. In this model, DES is induced by exposure of the animals to general anesthesia, which diminishes the production of basal and reflex tears and decreases the stability of the tear film thereby inducing common signs of the disease [4,28]. In a more recent study, we adopted a quantitative UPLC-MS/MS-based approach to identify and characterize baseline patterns of lipid mediators in the TF of healthy rabbits and demonstrated that they did not differ significantly from that of human TF [29]. Here, we employed these approaches to characterize TF lipidomic changes in DES, focusing on PUFAs, oxylipins and phospholipid derivatives contributing to the regulation of inflammation, and to find correlations between these changes and the pathophysiological and biochemical signs of the disease. Based on the data obtained, we proposed a novel complex anti-inflammatory therapy, targeting the specific oxylipin-mediated mechanisms revealed in DES. Using subsequent morphological, biochemical and lipidomic studies, this therapy was found to markedly improve the corneal state in DES by selective suppression of its inflammatory component, without affecting tear production rates.

## 2. Experimental Section

### 2.1. Materials

Anesthetic preparation containing 50 mg/mL tiletamine and 50 mg/mL zolazepam was from Virbac (Carros, France). Xylazine hydrochloride was from Nita-Farm (Saratov region, Russia). Ultragrade Tris was from Amresco (Solon, OH, USA). Phosphate-buffered saline (PBS) was from Thermo Fisher Scientific (Waltham, MA, USA). Zileuton, DMSO, hemoglobin, luminol, hydrogen peroxide solution, and Trolox (6-hydroxy-2,5,7,8-tetramethylchroman-2-carboxylic acid) were from Sigma-Aldrich (St. Louis, MO, USA). Reagents for histological examination were from Biovitrum (Moscow, Russia). Bicinchoninic acid (BCA) assay kit was from Sigma-Aldrich. Glutathione peroxidase and superoxide dismutase assay kits were from Randox (Crumlin, UK). Tumor necrosis factor alpha (TNF-α) and interleukin 10 (IL-10) assay kits were from Immunotex (Stavropol, Russia). The Schirmer’s test tear strips were from Contacare Ophthalmics and Diagnostics (Vadodara, Gujarat, India.). The deuterated oxylipins standards 6-keto PGF1α-d4, TXB2-d4, PGF2α-d4, PGE2-d4, PGD2-d4, LTC4-d5, LTB4-d4, 5(S)-HETE-d8, 12(S)-HETE-d8, 15(S)-HETE-d8, Oleoyl Ethanolamide-d4, EPA-d5, DHA-d5, and AA-d8 were from Cayman Chemical (Ann Arbor, MI, USA). Solid-phase lipid extraction cartridge Oasis^®^ PRIME HLB was obtained from Waters, Eschborn, Germany. Other reagents and suppliers used in this study were from Sigma-Aldrich, Amresco, or Serva (Heidelberg, Germany) and were at least of reagent grade. All buffers and other solutions were prepared using ultrapure water.

### 2.2. Experimental Animals and Ethics Statement

The study involved a total of 102 healthy New Zealand white rabbits (6 months old, weight of 2.3 to 3 kg) purchased from a certified farm (Krolinfo, Moscow region, Russia). The rabbits were housed in individual cages (795 × 745 × 1776 mm^3^) under normal conditions (12 h light/12 h dark, 22–25 °C, 55–60% humidity) with free access to food and water. The daily monitoring of health status of all animals involved ophthalmological exam, inspection of habitus, derma and mucous membranes as well as monitoring of heart/respiratory rates and body temperature. No adverse events were observed during the course of the study. The humane euthanasia of the animals intended for histological analysis of the anterior segment of the eye was performed by an overdose of the anesthetic (for general anesthesia conditions, see next section). The eyeballs of the animals were enucleated postmortem. Otherwise, the animals were rehabilitated for three days and returned to the farm. The animals were treated according to the 8th edition “Guide for the Care and Use of Laboratory Animals” of the National Research Council and “Statement for the Use of Animals in Ophthalmic and Visual Research” of The Association for Research in Vision and Ophthalmology (ARVO). The study was approved by the Belozersky Institute of Physico-chemical Biology Animal Care and Use Committee (Protocol number 1/2016, 12 January 2016).

### 2.3. Experimental Model

The experiments were performed using a single-blind method. The rabbits were divided into 17 groups, 6 animals in each group (Table 1), and treated as described in Section 3. DES was induced by introducing rabbits into prolonged general anesthesia as described in previous studies [6,30]. Particularly, the animals were placed in prone position in a restraining device and subjected to repeating intramuscular injection of anesthetic preparation containing 1:2 mixture of 50 mg/mL tiletamine/zolazepam and 20 mg/mL xylazine hydrochloride to achieve continuous narcotic sleep for 6 h. For the anti-inflammation therapy, the animals received conjunctival instillations of the eye drops, consisting of either 50% dimethyl sulfoxide (DMSO) in normal saline (0.90% *w*/*v* of NaCl), or the same solution containing 0.5 mg/mL (0.05% (*w*/*v*)) zileuton. The eye drops were prepared under sterile conditions. Each of the formulations was administrated 1 drop 3 times daily in each eye for up to 7 days, starting from the day of DES induction.

### 2.4. Histological Analysis

Histological analysis of the cornea was performed essentially as described earlier [6,31,32]. Briefly, the eyeball was enucleated immediately postmortem and fixed in 10% neutral buffered formalin in phosphate buffer (pH 7.4) for 24 h at room temperature. The cornea and iris were trimmed out, dehydrated (absolute isopropanol, 7 portions × 5 h) and embedded in Histomix paraffin medium. Eight 3-micron thick serial nasotemporal cross-sections of each cornea were obtained and mounted on glass slides. The sections were then deparaffinized (xylene, 5 min, two times), hydrated, and stained with Carazzi’s hematoxylin and 0.5% eosin Y. Stained sections were dehydrated by 96% ethanol and xylene, cleared in BioClear tissue clearing agent, mounted into BioMount synthetic medium, and examined using Leica DM4000 microscope with Leica DFC Camera (Leica, Wetzlar, Germany) or Zeiss AxioVert.A1 microscope with Axiocam IC C5 camera (Carl Zeiss, Oberkochen, Germany). Processing of the microphotographs was performed using the AxioVision 8.0 and ZEN 2 lite ZEISS Microscope Software (Carl Zeiss, Oberkochen, Germany) and Adobe Photoshop CS6 Extended (Adobe Systems, San Jose, CA, USA) software.

### 2.5. Schirmer’s Test

Tear secretion in animals was measured without topical or general anesthesia according to the following procedure [6,30]. Schirmer’s test tear strips were placed under the lower eyelid the restrained animal for 5 min, and the length of the moistened paper (in mm) was recorded. The procedure was repeated at least three times, and the average values were calculated.

### 2.6. TF Collection

The collection of TF for analytical purposes was performed in separate groups of animals (Table 1). For biochemical analysis, TF was obtained according to the Schirmer’s test procedure, except for the strip was allowed to get moistened for exactly 20 mm. Fifteen millimeters-long fragments of the strip were then cut off, extracted with 150 μL of PBS for 30 min and the resulting solution was collected and stored until the biochemical analysis (see Section 2.7, Section 2.8 and Section 2.9) at −80 °C [6]. To collect TF samples for lipidomic analysis [29] (see Section 2.10 and Section 2.11), the strip was allowed to get moistened reaching exactly 15 mm, the procedure was repeated with 3 strips and 15 mm fragments of each strip were cut off, transferred to a plastic tube with 1 mL of anhydrous methanol containing 0.1% *v*/*v* BHT and stored at −80 °C.

### 2.7. Total Protein Concentration Measurement

The concentration of total protein in TF samples was measured using BCA assay. Colorimetric reaction was monitored with Synergy H4 Hybrid plate reader (Biotek, Winooski, VT, USA). The calibration curve was plotted using standard solutions of bovine serum albumin in PBS.

### 2.8. Total Antioxidant Activity Analysis

The total antioxidant activity in TF was analyzed using hemoglobin/H_2_O_2_/luminol system [6,33,34]. Briefly, the TF samples were diluted 1:4 by PBS and added to the reaction mixture, containing 0.01 mM luminol and 0.5 mM hemoglobin. The reaction was initiated in the presence of 6 μM H_2_O_2_ and chemiluminescence of the samples was monitored every one second for 10 min using Glomax-Multi Detection System luminometer (Promega, Madison, WI, USA). The results were calculated in the Trolox equivalent after measuring chemiluminescence of standard solutions, containing 1–8 μM Trolox in PBS.

### 2.9. Antioxidant Enzymes Activity and Cytokine Concentration Analysis

The activities of superoxide dismutase and glutathione peroxidase and the contents of TNF-α and IL-10 in TF samples were measured without their additional dilution using the respective commercially available kits. The colorimetric reactions were monitored using Synergy H4 Hybrid Reader (Biotek, Winooski, VT, USA).

### 2.10. Lipid Extraction

The extraction of lipids for UPLC-MS/MS analysis was performed exactly as described in our resent study [29]. Briefly, TF samples were mixed with 2 ng of deuterated internal standard solutions and centrifuged (12,000× *g*, 3 min). The supernatants were mixed with 0.1% acetic acid (6 mL) and loaded onto solid-phase lipid extraction cartridge, which was washed with 15% methanol, 0.1% formic acid (2 mL), and the lipids were eluted with 500 μL of anhydrous methanol and subsequently with 500 μL of acetonitrile. The obtained TF extracts were concentrated by evaporation, reconstituted in 50 μL of 90% methanol and stored at −80 °C.

### 2.11. UPLC-MS/MS Analysis

TF lipids were analyzed in the obtained extracts using 8040 series UPLC-MS/MS mass spectrometer (Shimadzu, Japan) in multiple-reaction monitoring mode at a unit mass resolution for both the precursor and product ions [29,35]. The lipids separation was performed by reverse-phase UPLC on Phenomenex C8 column (0.4 mL/min, sample temperature of 5 °C). The compounds were eluted with 10–95% acetonitrile gradient in 0.1% (*v*/*v*) formic acid and subjected to MS analysis using electrospray ionization (in both positive and negative ion modes). The molecular ions were fragmentized by collision-induced dissociation in the gas phase and analyzed by tandem (MS/MS) mass spectrometry. The selected lipids were identified and quantified by comparing their mass-spectrometric and chromatographic data with those obtained for the corresponding oxylipins standards (see Section 2.1.) using Lipid Mediator Version 2 software (Shimadzu, Japan).

### 2.12. Statistical Analysis

The line charts in the figures represent mean ± SEM. Statistical significance was assessed using the repeated measure ANOVA (for analysis within the groups) and one way ANOVA (for analysis between the groups) with post-hoc pairwise comparisons using Tukey’s test. The probability of less than 0.05 was considered significant.

## 3. Results

### 3.1. Dynamics of Inflammation in DES: Tear Production and Morphological Changes in the Cornea

To characterize the inflammatory responses associated with DES, a control group and four experimental groups of animals were created (groups 1–5, Table 1). To induce DES, the animals within all the experimental groups were exposed to six hours of general anesthesia in accordance with the approach developed in our previous study [6]. Using this setup, we assessed the dynamics of the pathological process in DES, focusing on its inflammatory component. To this end, we performed a histopathological examination of corneas obtained from animals euthanized immediately after anesthesia (at the sixth hour), as well as on days one, three and seven. To complete DES characterization, the intensity of tear production was analyzed in a separate group of animals (group 6, Table 1) using a standardized Schirmer’s test.

**Figure 1:**
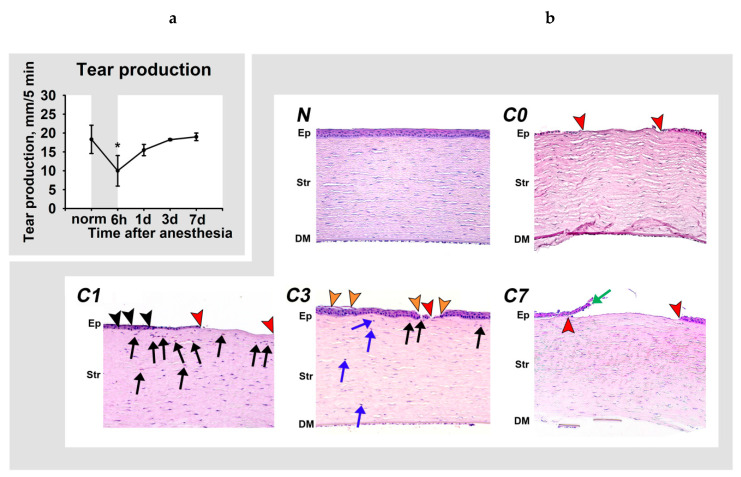
Dynamic changes in tear secretion and corneal morphology in general anesthesia-induced DES. (**a**) The results of standardized Schirmer’s tests performed in animals at 6th hour, 1st day, 3rd day or 7th day after their exposure to 6-h general anesthesia. ∗ *p* < 0.05 compared with the values measured in the control group. (**b**) Representative microscopic images of hematoxylin and eosin-stained cross-sections of the corneas obtained at 6th hour (C0), 1st day (C1), 3rd day (C3) and 7th day (C7) after exposure of the animals to general anesthesia. The image of the normal cornea is also presented (N). Stromal denudation (complete absence of the corneal epithelium) areas (red arrowheads), loci of desquamation/destruction of the epithelial layer (orange arrowheads), and sites of reepithelialization (green arrows; the detachment of the new epithelium from the stroma represents an artifact stemmed from histological processing) are indicated. The inflammatory changes include granulocytic infiltration of the corneal epithelium (intraepithelial granulocytes; black arrowheads) and stroma (black arrows), and postinflammatory signs of regeneration (activation of stromal keratocytes) at the ex-sites of the infiltration (blue arrows). Ep: epithelium; Str: stroma; DM: Descemet’s membrane and endothelium. Hematoxylin and eosin staining, magnification 200×. The central areas of the cornea are presented. Gaps, cracks and folds of preparations are of an artificial origin.

It was found that tear production decreased two-fold immediately after anesthesia and was completely restored only on day three (Figure 1a). These changes were associated with pronounced morphological changes in the cornea, involving denudation of the corneal stroma, which had already appeared during the period of narcotic sleep, as a result of a large number of deaths of the corneal epithelial cells (Figure 1b, C0). The denudation was preserved focally on day one (Figure 1b, C1). On the remainder of the surface, the epithelium was non-homogenous, consisting of different numbers of layers. Notably, at this stage, the epithelium was abundantly infiltrated by inflammatory cells, mainly granulocytes. Such infiltrates were even found in the areas, where its structure was not significantly altered. The corneal stroma also contains a granulocyte infiltrate. Moderate areas of denudation and multiple epithelial lesions, together with the signs of inflammatory infiltration in the stroma but without intraepithelial infiltration, remained on day three (Figure 1b, C3). In addition, at this time-point, the stroma contained activated keratocytes and exhibited remodeling processes. The signs of inflammation were not entirely resolved until the seventh day after DES induction (Figure 1b, C7). Meanwhile, sporadic areas of denudation remained until day seven, when they were surrounded by reepithelialization rolls along the edges, indicating a developed healing process.

We concluded that the pathological process in our DES model involved ocular surface inflammation, reaching a maximum on day one after anesthesia, which was accompanied by a decline in tear production and prolonged corneal damage.

### 3.2. Dynamics of Inflammation in DES: Biochemistry and Lipidomics of the Tear Fluid

**Figure 2:**
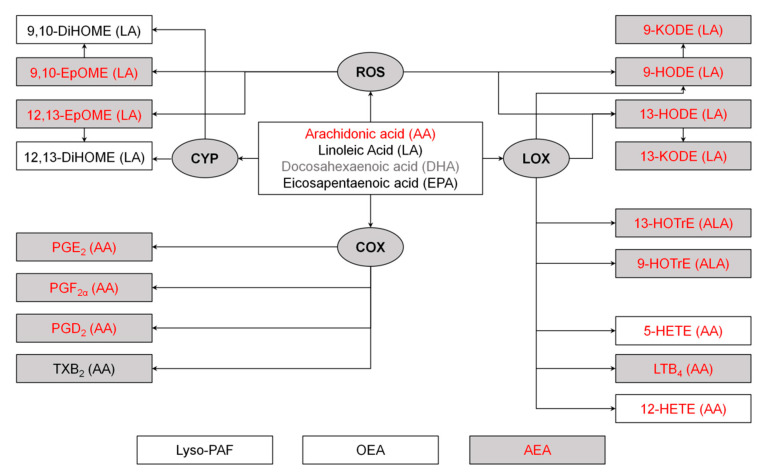
Mechanisms of biosynthesis of lipid mediators. Metabolites of arachidonic acid (AA), linoleic acid (LA) and alpha-linolenic acid (ALA) are divided into the groups synthesized via lipoxygenase (LOX), cyclooxygenas (COX), cytochrome P450 monooxygenase (CYP) or reactive oxygen species (ROS) dependent pathways. The other abbreviations are as follows: AEA, N-arachidonoylethanolamine; DHA, docosahexaenoic acids; 9,10-DiHOME, 9,10-dihydroxyoctadecamonoenoic acid; 12,13-DiHOME, 12,13-dihydroxyoctadecamonoenoic acid; EPA, eicosapentaenoic acid; 9,10-EpOME, 9,10-epoxyoctadecamonoenoic acid; 12,13-EpOME, 12,13-epoxyoctadecamonoenoic acid; 9-HODE, 9-hydroxyoctadecadienoic acid; 13-HODE, 13-hydroxyoctadecadienoic acid; 9-KODE, 9-oxo-octadecadienoic acid; 13-KODE, 13-oxo-octadecadienoic acid; LTB4, leukotriene B4; lyso-PAF, lyso-platelet-activating factor; OEA, oleoylethanolamine; PGD2, prostaglandin D2; PGE2, prostaglandin E2; PGF2α, prostaglandin F2α. The mediators altered in DES and affected by the proposed anti-inflammatory therapy (see below) are indicated in red and gray colors, respectively.

To understand the mechanisms underlying the inflammatory reactions observed on the ocular surface, we next examined biochemical markers of the associated inflammatory pathways, focusing on lipid mediators (Figure 2). The analysis was performed on TF, which is known to be responsible for nourishing the cornea and maintaining the immune responses of the ocular surface [29]. TF was collected using Schirmer’s strips in experimental group 8 (Table 1), following the aforementioned timeline and analyzed by a high-performance, quantitative mass-spectrometric approach, developed in our recent studies [29]. In parallel, TF samples (group 7, Table 1) were analyzed for total protein content, characteristic proinflammatory (TNF-α) and anti-inflammatory (IL-10) cytokines and antioxidant molecules (total antioxidant activity (low molecular weight antioxidants), superoxide dismutase and glutathione peroxidase), which were previously characterized as markers of anesthesia-induced DES [6].

**Figure 3:**
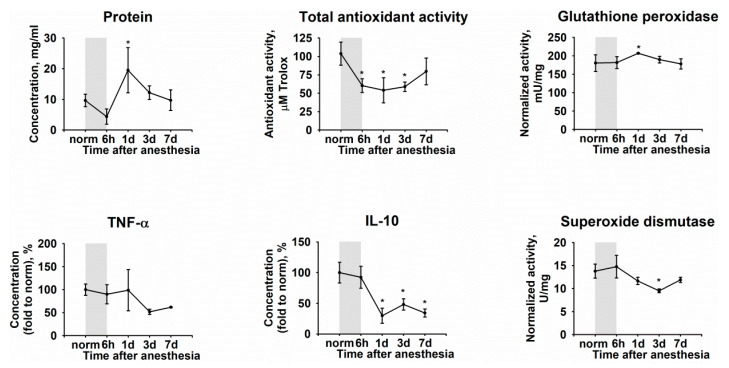
Dynamic changes in biochemical properties of TF in DES. The animals were exposed to general anesthesia for 6 h (shown as gray box). TF samples were collected before (norm) and immediately after (6 h) the general anesthesia as well as on 1, 3, and 7 day post-exposure. The samples were analyzed for total protein, TNF-α, IL-10, as well as total antioxidant activity (in Trolox equivalent) and activity of glutathione peroxidase and superoxide dismutase using the respective assays. * *p* < 0.05 as compared to parameters of TF of the intact (control) animals.

It was noted that the pathological process reflected in TF as a rapid decline of the total antioxidant activity and moderate upregulation of glutathione peroxidase (day one) as well as a more delayed suppression of superoxide dismutase, manifested on day three (Figure 3). The observed alterations were accompanied by inflammatory changes, such as an increase in total protein concentration and a decrease in IL-10 content, without having a significant effect on TNF-α. Importantly, these changes were most pronounced on day one, which concurred with histological findings (see Figure 1).

**Figure 4:**
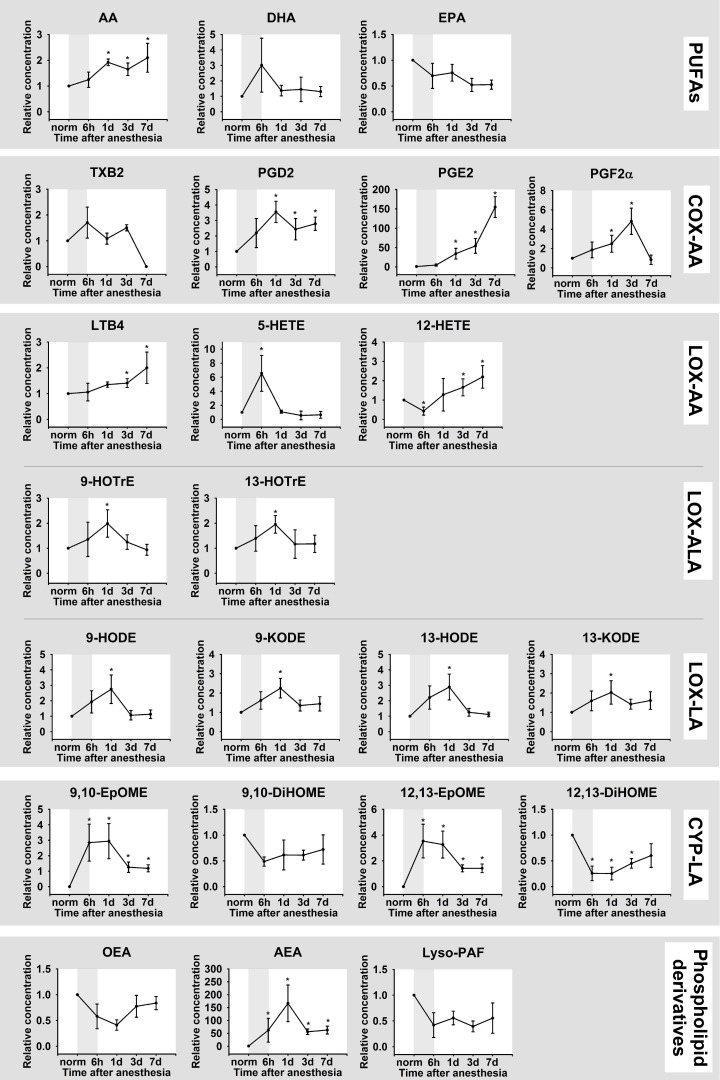
Dynamic changes in TF content of lipid mediators in DES. The animals were exposed to general anesthesia for 6 h (shown as gray box). TF samples were collected before (norm) and immediately after (6 h) the general anesthesia as well as on 1, 3, and 7 day post-exposure. The concentrations of the lipid mediators (phospholipid derivatives, PUFAs and oxylipins) in TF were measured using quantitative UPLC-MS/MS analysis. * *p* < 0.05 as compared to the parameters of TF of the intact (control) animals. The identified oxylipins are divided into subgroups according to their precursors (AA, ALA, LA, phospholipids) and major biosynthetic pathways, involving COX, LOX, or CYP.

A UPLC-MS/MS analysis of TF samples revealed a total of 23 lipid mediators, including three PUFAs (AA, DHA and EPA), 18 oxylipins and three phospholipid derivatives (OEA, AEA, Lyso-PAF) (Figure 4; full names and biosynthesis pathways for all lipids detected in the study are presented in Figure 2). The identified oxylipins were assigned to five subgroups, namely cyclooxygenase (COX)-dependent derivatives of AA (PGD2, PGE2, PGF2α, TXB2), cytochrome p450 (CYP)-dependent derivatives of LA (9,10-DiHOME/9,10-EpOME and 12,13-DiHOME/12,13-EpOME), as well as lipoxygenase (LOX)-dependent derivatives of AA (5-HETE, 12-HETE, and LTB4), LA (9-HODE/9-KODE and 13-HODE/13-KODE) and ALA (9-HOTrE, 13-HOTrE). The detected lipid mediators exhibited different behavior in DES. Thus, the most prolonged change in TF content was observed for AA and its COX-dependent (PGD2, PGE2, PGF2α) and LOX-dependent (LTB4, 5-HETE, 12-HETE) products, most of which exhibited sustained growth until the seventh day after the anesthesia. The significant increase in LTB4 was accompanied by an increase and a subsequent decrease in the TF content of its precursor 5-HETE. In turn, the LOX/oxidative stress-dependent derivatives of LA (9-HODE/9-KODE and 13-HODE/13-KODE), LOX-dependent derivatives of ALA (9-HOTrE, 13-HOTrE), CYP derivatives of LA (9,10-EpOME and 12,13-EpOME) as well as the phospholipid derivative AEA demonstrated a short-term increase on day one indicating the critical significance of this time-point as the peak of the oxidative and inflammatory response. Finally, no significant DES-dependent changes were observed in the case of DHA and EPA, as well as OEA and Lyso-PAF.

Considered together, these data demonstrate that anesthesia-induced DES is associated with oxidative stress and inflammatory response, with the latter being governed mainly by AA and its derivative oxylipins, namely 5-HETE/LTB4 (5-LOX products) and 12-HETE (12-LOX product) as well as COX-dependent prostaglandins (PGD2, PGE2, PGF2α).

### 3.3. Selective Targeting of Inflammation in DES: Rationale for the Drug Formulation

The obtained lipidomic data indicated that ocular inflammation in DES was governed mainly by two pathways, which depend on AA release and involve its processing by 5/12-LOX and COX. Thus, its targeting may include compounds dampening these pathways. The common COX inhibitors, NSAIDs, are efficacious in relieving different forms of ocular inflammation, but they can exacerbate DES by causing severe side effects [25,26]. Therefore, we focused on alternative methods of treatment, such as targeting LOX-dependent pathways. For this purpose, we chose zileuton (N-(1-Benzo(b)thien-2-ylethyl)-N-hydroxyurea), an anti-asthmatic drug, selectively inhibiting 5-LOX and thereby suppressing the generation of leukotrienes, including LTB4 [36]. An important benefit of zileuton is that it also inhibits AA release and, consequently, downregulates prostaglandins [37].

Given that zileuton (a low water-soluble drug) had never been employed in ophthalmology, a new formulation was developed for its administration in the form of eye drops, containing dimethyl sulfoxide (DMSO). DMSO was selected as it is low toxic [38,39], high tissue permeable, and facilitates the penetration of other compounds through biological membranes [40,41]. The use of up to 50% DMSO was recognized as not causing any adverse reactions upon topical administration in the eye [38,42]. Since maximum solubility of zileuton in 50% solution of DMSO in PBS was reported to be 0.5 mg/mL [43], we settled on the formulation of complete eye drops, consisting of 0.5 mg/mL zileuton in normal saline, containing 50% DMSO. According to the results of clinical examinations performed by an experienced veterinarian (co-author of this study), this dosage was well tolerated by both healthy rabbits and the animals with DES. Thus, no signs of local (i.e., in the cornea, conjunctiva, iris or eyelids) or systemic reactions were registered. It should be emphasized that the formulation was not regarded as final, but it was approved for primary verification of the proposed anti-inflammatory approach.

### 3.4. Selective Targeting of Inflammation in DES: A Morphological Study

To trial the suggested anti-inflammatory therapy, DES was induced in six experimental groups of animals (groups 6–11, Table 1), which subsequently received instillations of the complete zileuton/DMSO eye drops (zileuton groups) or 50% DMSO in normal saline (DMSO groups). One drop of each of these forms was administrated three times daily in each eye for up to seven days and the animals were euthanized on days one, three and seven following the anesthesia for histological analysis.

**Figure 5:**
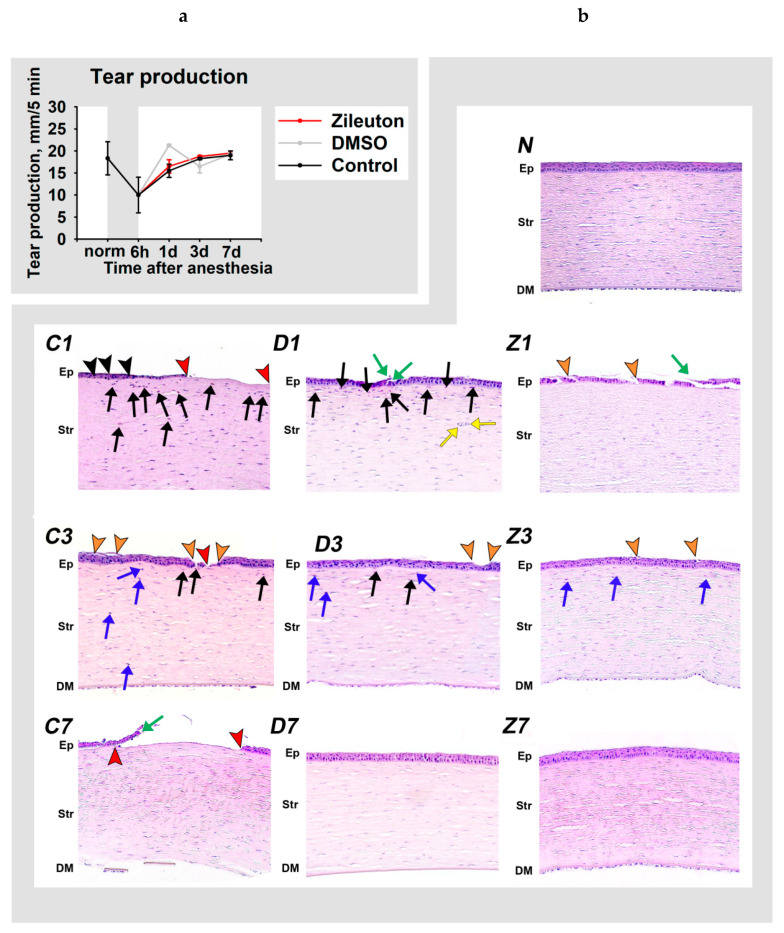
Dynamic changes in tear secretion and corneal morphology in DES in the course of anti-inflammatory therapy. The animals were exposed to general anesthesia for 6 h (shown as gray box) and subsequently received instillations of the eye drops containing 50% DMSO (D1, D3, D7) or 50% DMSO with 0.5% zileuton (Z1, Z3, Z7) for 7 days. (**a**) The results of standardized Schirmer’s tests performed at 6th hour, 1st day, 3rd day or 7th day after the exposure. (**b**) Representative microscopic images of hematoxylin and eosin-stained cross-sections of the corneas at 1st (D1, Z1), 3rd (D3, Z3) and 7th (D7, Z7) day after exposure. The image of the normal cornea is also presented (N). Stromal denudation (complete absence of the corneal epithelium) areas (red arrowheads), loci of desquamation/destruction of the epithelial layer (orange arrowheads), and sites of reepithelialization (green arrows) are indicated. The inflammatory changes include granulocytic infiltration in the corneal epithelium (intraepithelial granulocytes; black arrowheads) and stroma (black arrows), neovascularization (yellow arrows), and postinflammatory signs of regeneration (activation of stromal keratocytes) at the ex-sites of the infiltration (blue arrows). Ep: epithelium; Str: stroma; DM: Descemet’s membrane and endothelium. Hematoxylin and eosin staining, magnification 200×. The central areas of the cornea are presented. Gaps, cracks and folds of preparations are of an artificial origin.

Schirmer’s tests performed on similar groups of animals receiving the same instillations of zileuton/DMSO or DMSO (groups 12 and 15, Table 1), revealed no effect of the therapy on tear production (Figure 5a). Yet, already on the first day after the anesthesia, the corneas of zileuton groups exhibited pronounced histological signs of epithelial recovery, such as reepithelization rolls (Figure 5b, Z1). The area of denudation was minimal, as single-layer epithelium covered a significant part of the surface, which was drastically in contrast to the untreated samples. Importantly, at this time-point, the cornea exhibited almost no inflammatory infiltrate both in epithelium and stroma. On day three, the epithelium was restored across most of the cornea: the stromal denudation areas were rare and their size did not exceed single cells (Figure 5b, Z3). The newly formed epithelium had a thickness of several layers, although it did not reach the full-size characteristic of normal tissue. These samples exhibited no edema and acute inflammation, but there were signs of the completed inflammatory process and subsequent regeneration, such as an increased number of activated keratocytes at the corresponding sites. Finally, on day seven, the cornea of the rabbits which received zileuton (Figure 5b, Z7) was indistinguishable from the tissue of the healthy animals (Figure 5b, N).

Interestingly, some of these effects can be attributed to DMSO, as animals of the DMSO group also exhibited accelerated recovery from the anesthesia-induced lesions. Thus, on day one after anesthesia, their cornea contained far fewer denudation areas and signs of intraepithelial infiltration by granulocytes (Figure 5b, D1). However, on days one to three, its stroma was still infiltrated by granulocytes (Figure 5b, D1, D3), whereas in the zileuton group, these infiltrations were completely absent (Figure 5b, Z1, Z3). Furthermore, in the DMSO group, the stroma contained newly formed capillaries (signs of neovascularization), characteristic of keratitis (Figure 5b, D1). Although these animals exhibited early signs of regeneration, these were less prominent than in the zileuton group. Overall, DMSO enhanced corneal healing and produced a moderate anti-inflammatory effect, while zileuton effectively reduced stromal infiltration by granulocytes, neovascularization and other manifestations of acute inflammation.

Overall, we concluded that the treatment using zileuton/DMSO-containing eye drops can markedly improve the corneal state in DES, by selective suppression of its inflammatory component without affecting tear production rates.

### 3.5. Selective Targeting of Inflammation in DES: Biochemistry and Lipidomics of the Tear Fluid

**Figure 6:**
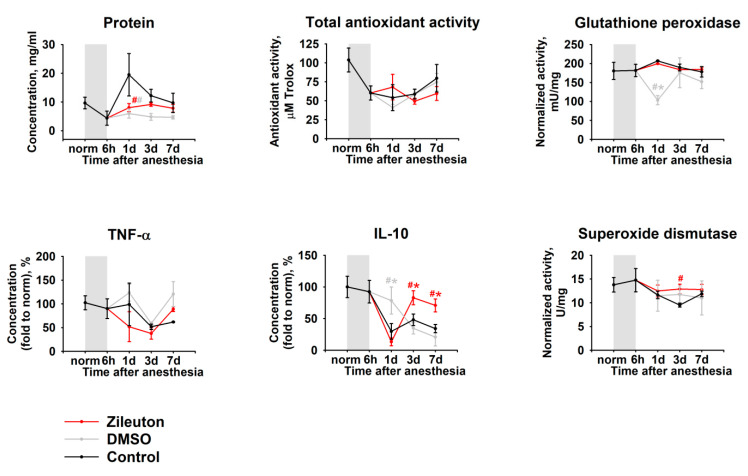
Dynamic changes in biochemical properties of TF in DES in the course of anti-inflammatory therapy. The animals were exposed to general anesthesia for 6 h (shown as gray box) and subsequently received instillations of the eye drops containing 50% DMSO (gray) or 50% DMSO with 0.5% zileuton (red), 1 drop 3 times daily in each eye for 7 days. TF samples were collected before (norm) and immediately after (6 h) general anesthesia as well as on 1, 3, and 7 day post-exposure. The samples were analyzed for total protein, TNF-α, IL-10, total antioxidant activity (in Trolox equivalent) and activity of glutathione peroxidase and superoxide dismutase using the respective assays. # *p* < 0.05 as compared to parameters of TF of the untreated animals with DES (black). * *p* < 0.05 as compared to parameters of TF of the animals from the DMSO or zileuton group.

To address the mechanisms underlying the observed therapeutic effects, we next analyzed alterations in biochemical properties and the lipid content of TF, associated with zileuton/DMSO treatment. To this end, TF was collected in animals from two DES groups (groups 13–14 and 16–17, Table 1) receiving either complete eye drops (zileuton groups) or DMSO alone (DMSO groups). The therapy using zileuton/DMSO had almost no impact on the antioxidant activity of the TF but prevented the increase in total protein concentration in the TF (Figure 6). These effects could be associated with DMSO activity, as a similar picture was observed in the DMSO group. Yet only the therapy with eye drops containing zileuton upregulated the anti-inflammatory cytokine IL-10 and attenuated the decline in activity of the superoxide dismutase.

**Figure 7:**
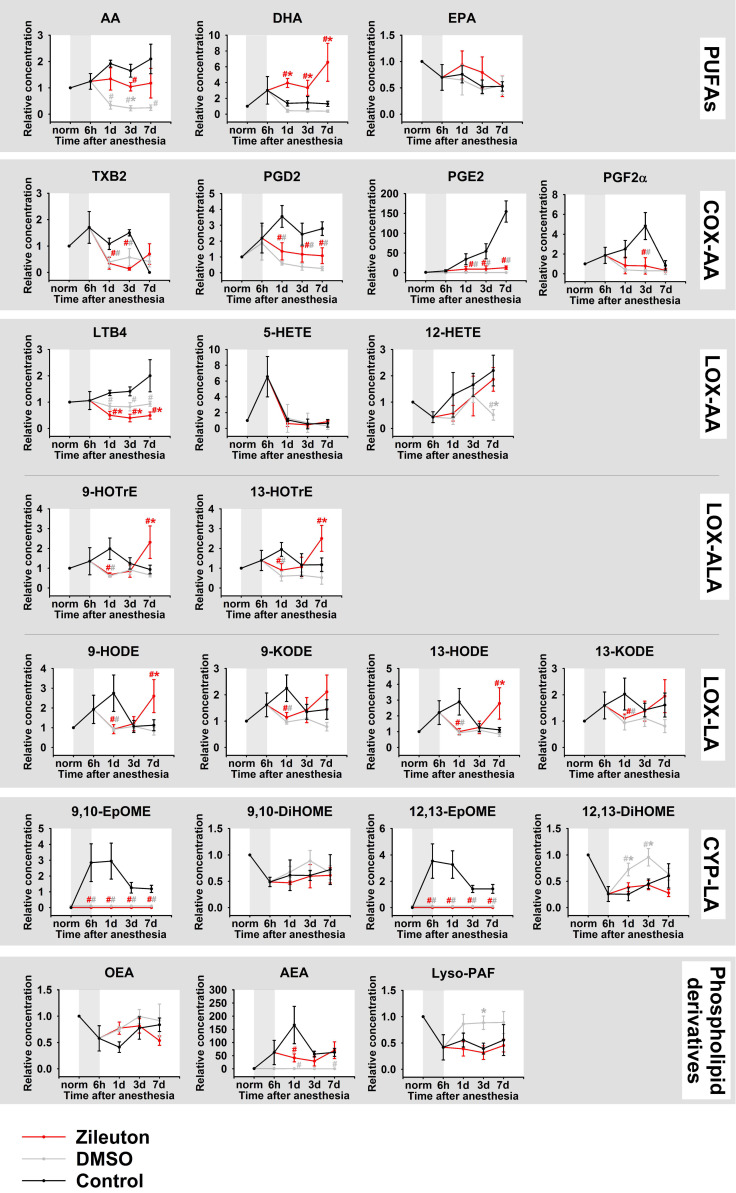
Dynamic changes in TF content of lipid mediators in DES in the course of anti-inflammatory therapy. The animals were exposed to general anesthesia for 6 h (shown as gray box) and subsequently received instillations of the eye drops containing 50% DMSO (gray) or 50% DMSO with 0.5% zileuton (red), one drop three times daily in each eye for 7 days. TF samples were collected before (norm) and immediately after (6 h) the general anesthesia as well as on 1, 3, and 7 day post-exposure. The concentrations of the lipid mediators (phospholipid derivatives, PUFAs and oxylipins) in TF were measured using quantitative UPLC-MS/MS analysis. # *p* < 0.05 as compared to parameters of TF of the untreated animals with DES (black). * *p* < 0.05 as compared to parameters of TF of the animals from the DMSO or zileuton group.

These findings were consistent with the results of the UPLC-MS/MS analysis of lipid mediators of TF in animals of the same groups, confirming the antioxidant and pronounced anti-inflammatory activity of the proposed therapy (Figure 7). Thus, zileuton/DMSO treatment prevented an increase in AA and its derivatives, prostaglandins (PGD2, PGE2, PGF2α) and LTB4, whereas the contents of 5-HETE and 12-HETE were much less affected. In addition, the therapy suppressed short-term elevations in the content of LOX/oxidative stress-dependent derivatives of LA (9-HODE/9-KODE and 13-HODE/13-KODE), LOX-dependent derivatives of ALA (9-HOTrE, 13-HOTrE), CYP derivatives of LA (9,10-EpOME and 12,13-EpOME) and the phospholipid derivative AEA.

Interestingly, some of these effects were similar among the zileuton and DMSO groups, thereby demonstrating for the first time the broad anti-inflammatory activity of DMSO. However, LTB4, the main product of 5-LOX, was faster and more potently downregulated in the zileuton group and its level was even less than among healthy animals. Furthermore, zileuton treatment generally increased the TF content of anti-inflammatory mediator DHA, whereas DMSO had no effect in this regard. It should be added that the animals from the zileuton group also exhibited increased content of oxylipins 9-/13-HOTrE and 9-/13-HODE on the seventh day apparently representing delayed or compensatory response on the therapy.

Overall, based on the data obtained, we can conclude that the high efficacy of the proposed complex therapy is a result of the synergetic action of two components of the eye drops, namely the general healing property of DMSO and the more specific anti-inflammatory effects of zileuton.

## 4. Discussion

Our study represents the first comprehensive characterization of inflammatory mechanisms in DES mediating by PUFAs and their derivatives, oxylipins, which was performed in a time-dependent manner, considering the alterations in tear production rates, as well as the pathophysiological and biochemical (oxidative stress, cytokine response) signs of the disease. Importantly, it is these mechanisms that are most commonly impacted by the currently approved anti-inflammatory drugs (such as dexamethasone or NSAIDs) and represent the prospective targets for novel specific therapies. Generally, the nomenclature of inflammatory mediators in rabbit tears identified in this study (23 lipids), accords with our recent findings regarding human TF [29]. A number of attempts have been made to examine alterations in the TF content of PUFAs/oxylipins in DES, both in animal and human studies, but most of them have focused on single mediators, such as three PUFAs (AA, DHA and EPA) and two prostaglandins (PGE2 and PGD2). Thus, it was found that the ratio of ω-6 (AA) to ω-3 (DHA + EPA) fatty acids is increased in DES patients [21], which concurs with our observations (see Figure 3). Furthermore, a number of works reported DES-associated growth of the TF level of PGE2, which is recognized as a hallmark of the disease [21,22,23,44]. Consistently, in our experiments, PGE2 exhibited the most pronounced elevation among the identified mediators (see Figure 3). To date, the most detailed study of lipid mediators in DES has focused on eicosanoids, which were profiled in the TF of healthy individuals, in comparison with MGD patients, by means of an MS-based approach [45]. The reliable disease-associated increase was found in relation to six compounds, including 5-HETE and LTB4, which were similarly increased in our DES model. Furthermore, our results complement these data well, since we detected a significant increase in two more prostaglandins, PGD2 and PGF2α, as well as 12-HETE. Overall, taking into account our findings and the existing literature data, we can conclude that an inflammatory response in DES is governed mainly by AA and its derivative oxylipins 5-HETE and LTB4 (5-LOX products), 12-HETE (12-lipoxigeanse product) as well as PGD2, PGE2 and PGF2α (cyclooxygenase products). Notably, in our experiments, most of the aforementioned lipids demonstrated a prolonged increase not reaching their maximum even after seven days of observation, which correlates with their permanent elevations in human DES patients, exhibiting chronic inflammation.

It should be added that among the changes in AA derivatives, there was an increase in AEA (anandamide), a product of the non-oxidative metabolism of AA-containing phospholipid N-arachidonoyl phosphatidylethanolamine. AEA is an endocannabinoid, exhibiting proinflammatory activity as it was found to exacerbate endotoxin-induced uveitis, enhancing the clinical score of intraocular inflammation and increasing the amount of leukocyte and protein concentration in the aqueous humor [46]. Our results represent the first indication of the participation of AEA in the inflammatory response associated with DES, recognizing this compound as one of the promising targets for use in an anti-inflammatory therapy for the disease.

In contrast to AA and its products, six LA derivatives, namely 9-HODE/9-KODE, 13-HODE/13-KODE, and 9,10-EpOME/12,13-EpOME, exhibited a pronounced but short-term increase, manifested only on day one after general anesthesia. In fact, this time-point represents a critical period not only for the inflammatory reaction but also for oxidative responses in our DES model. Thus, on day one, the most pronounced granulocytic infiltration in the corneal stroma, maximal protein concentration in TF and the most prominent cytokine alterations were accompanied by minimal antioxidant activity in TF, indicating the development of oxidative stress. We suggest that increased levels of 9-HODE/9-KODE and 13-HODE/13-KODE may reflect non-enzymatic oxidation of LA by ROS. Indeed, these compounds are well-recognized markers of oxidative stress [47,48]. Similarly, biosynthetic pathways for 9,10-EpOME and 12,13-EpOME, which are produced by neutrophils and macrophages and mediate different inflammatory effects, may involve non-enzymatic oxidation [20,49,50]. It was suggested that inflammatory changes on the ocular surface in DES are related to chronic oxidative stress, accompanied by reduced local antioxidant activity [51,52]. For instance, superoxide dismutase downregulation was shown to be associated with inflammation in the lacrimal glands and decreased tear secretion [53], and we observed a decrease in superoxide dismutase activity in DES. Thus, the quenching of the oxidative stress by topical administration of antioxidants may represent a powerful strategy in the treatment of the disease (including its inflammatory component), which was confirmed in our recent study [6].

In addition to AA and LA derivatives, we detected two products of ALA, 9-HOTrE and 13-HOTrE, to be elevated in the acute phase of inflammation (day one). Notably, 9-HOTrE can be produced from ALA by 5-LOX and glutathione peroxidase [54]. Moreover, these enzymes can catalyze the production of AA into 9-HODE [54], another oxylipin increased in the acute phase. In our biochemical experiments, glutathione peroxidase was found to be moderately upregulated in day one, which supports the importance of the aforementioned cascades in DES. Thus, in summary, we detected four LOX-5-dependent oxylipins elevated in the early (9-HODE and 9-HOTrE) and late (5-HETE and LTB4) phases of anesthesia-induced DES. Based on these data, we proposed that the inflammatory response, associated with the disease, can be efficiently treated by the topical administration of zileuton, the only specific inhibitor of 5-LOX, approved by the US FDA [36,55]. The choice of zileuton was especially justified, as it was previously shown to inhibit the release of AA and, consequently, to downregulate prostaglandins [37], which, according to our data, contributed most to the development of prolonged inflammation in DES. Zileuton is a low water-soluble drug which has never been employed in ophthalmology, to the best of our knowledge. Yet, topical administration of zileuton (500 µg) dissolved in 100% DMSO was previously employed in animal studies for the treatment of ear edema [56]. In this study, we used eye drops containing 0.5 mg/mL solution of zileuton in 50% DMSO. Such concentration of DMSO was recognized as not causing any adverse reactions upon topical administration in the eye [38,42]. Indeed, according to our observations, the resulting eye drops were generally well tolerated by the animals. It should be noted that the described composition of the eye drop was approved only for primary verification of the proposed anti-inflammatory approach in this study. Further studies are required for careful optimization of the dosage of the proposed zileuton/DMSO-based prodrug.

The suggested treatment produced an unexpectedly prominent therapeutic benefit, preventing granulocytic infiltration and other signs of inflammation, and accelerating corneal healing by at least three days. The underlying mechanisms involved selective inhibition of inflammation without affecting the antioxidant activity and tear-producing rates. Interestingly, some of the observed benefits can be attributed to DMSO. Indeed, this compound was previously shown to exhibit its own therapeutic activity in the eye by facilitating corneal healing and exhibiting anti-inflammatory effects [57,58,59,60]. The mechanisms underlying these benefits may be related to the downregulation of PGE2 [61]. A similar effect of DMSO was observed in our model (see Figure 6). Yet, we have found that DMSO suppressed several other pathways indicating its broader biological action. For example, it significantly reduced the activity of glutathione peroxidase, which may partially underlie the suppression of 9-HODE and 9-HOTrE content in TF [54], seen in our experiments. Downregulation of 9-HODE and 13-HODE may inhibit monocyte activation, reduce pain syndrome and produce other therapeutic effects in DES [62,63]. Currently, the intravesical instillation of 50% aqueous solution DMSO (RIMSO-50^®^) is approved by the FDA for the treatment of interstitial cystitis. Furthermore, DMSO is used as a cosolvent in several other FDA-approved products, including Onyx^®^ (injection into brain arteriovenous malformation), Viadur^®^ (subcutaneous implantation) and Pennasid^®^ (topical administration). Although in early studies the ocular effects of DMSO were considered contradictory and there was no systematic examination of DMSO-based formulations in ophthalmic clinical trials, today, growing evidence confirms the feasibility of this compound as a cosolvent in topical medication for the treatment of a wide range of ophthalmological conditions, including superficial keratitis, blepharoconjunctivitis, blepharitis and glaucoma [64,65,66,67]. Considered together, these data justify the future trialing safety and efficacy of a 50% DMSO-based solution of zileuton.

Despite the prominent healing activity of DMSO, the introduction of zileuton has led to a substantial improvement in the therapeutic benefit of the eye drops. Thus, according to our histological data, in the DMSO group, the signs of inflammation, such as infiltration by granulocytes and neovascularization, remained one to three days after general anesthesia, whereas in the zileuton group, these signs were absent (see Figure 5b). Indeed, zileuton produced additional, more specific anti-inflammatory effects. The first specific effect was the pronounced upregulation of IL-10, the anti-inflammatory cytokine previously found to be associated with DES [68]. Interestingly, a similar effect of zileuton against IL-10 was demonstrated in other inflammation-related disorders [69]. The second specific effect of zileuton was the downregulation of the proinflammatory mediator, LTB4. This oxylipin was shown to govern ocular inflammation, being secreted by the corneal, conjunctival and Meibomian gland epithelium in response to lipopolysaccharide action [70]. A specific increase in the TF concentration of LTB4 was also induced by contact lens wearing, a common ethological factor in DES [71]. Given that LTB4 was established as a key factor in recruiting effector T cells [72,73], its downregulation by zileuton might specifically suppress the T-cell response in DES (see Introduction section). The third specific effect of zileuton was the pronounced upregulation of the anti-inflammatory mediator, DHA. Previously, the deficiency of DHA and EPA was found to correlate with the clinical manifestations of DES [21]. Consistently, the daily supplementation of these ω-3 PUFAs was found to reduce or reverse the symptoms of DES in different species including humans [74,75,76]. DHA is a well-recognized precursor of proresolving lipid mediators, controlling epithelial wound healing, inflammatory cell migration and nerve regeneration [21]. Thus, the treatment with zileuton-containing eye drops might affect the resolution of the inflammatory response in DES by promoting the release of DHA and its derivative resolvins.

Overall, we can conclude that the high efficacy of the proposed complex therapy resulted from the synergetic action of its components, DMSO and zileuton. The general healing activity of DMSO includes the downregulation of prostaglandins. Meanwhile, zileuton suppresses cytokine-dependent inflammatory mechanisms, apparently restrains the T-cell response via selective inhibition of LTB4 and promotes resolution pathways by upregulating DHA. Thus, these compounds act synergistically, encompassing virtually all aspects of the mechanism underlying the inflammatory response in DES. Based on these data, we suggest that the proposed therapeutic approach can be considered in future as one of the promising methods of DES treatment.

## Figures and Tables

**Table 1:** Parameters of the experimental groups.

Group	Treatment	Analysis	Time of Analysis
1	-	Histology	0 h (control)
2	-	Histology	6 h
3	-	Histology	1 d ^1^
4	-	Histology	3 d
5	-	Histology	7 d
6	-	Schirmer’s test	0 h, 6 h, 1 d, 3 d, 7 d
7	-	Biochemistry ^1^	0 h, 6 h, 1 d, 3 d, 7 d
8	-	Lipidomics ^2^	0 h, 6 h, 1 d, 3 d, 7 d
6	DMSO	Histology	1 d
7	DMSO	Histology	3 d
8	DMSO	Histology	7 d
9	DMSO/zileuton	Histology	1 d
10	DMSO/zileuton	Histology	3 d
11	DMSO/zileuton	Histology	7 d
12	DMSO	Schirmer’s test	1 d, 3 d, 7 d
13	DMSO	Biochemistry ^1^	1 d, 3 d, 7 d
14	DMSO	Lipidomics ^2^	1 d, 3 d, 7 d
15	DMSO/zileuton	Schirmer’s test	1 d, 3 d, 7 d
16	DMSO/zileuton	Biochemistry ^1^	1 d, 3 d, 7 d
17	DMSO/zileuton	Lipidomics ^2^	1 d, 3 d, 7 d

^1^ Measurement of total protein concentration, total antioxidant activity, antioxidant enzyme activity, and cytokine concentration in TF; ^2^ UPLC-MS/MS of lipid mediators (PUFAs, oxylipins and phospholipid derivatives) in TF; d: day.

## Abbreviations

AA	Arachidonic acid
AEA	N-arachidonoylethanolamine
ALA	Alpha-linolenic acid
BCA	Bicinchoninic acid
COX	Cyclooxygenases
CYP	Cytochrome P450 monooxygenases
DES	Dry eye syndrome
DHA	Docosahexaenoic acids
DMSO	Dimethyl sulfoxide
9,10-DiHOME	9,10-dihydroxyoctadecamonoenoic acid
12,13-DiHOME	12,13-dihydroxyoctadecamonoenoic acid
EPA	Eicosapentaenoic acid
9,10-EpOME	9,10-epoxyoctadecamonoenoic acid
12,13-EpOME	12,13-epoxyoctadecamonoenoic acid
9-HODE	9-hydroxyoctadecadienoic acid
13-HODE	13-hydroxyoctadecadienoic acid
IL-1	Interleukin-1
IL-10	Interleukin-10
IL-1β	Interleukin-1 beta
9-KODE	9-oxo-octadecadienoic acid
13-KODE	13-oxo-octadecadienoic acid
LTB4	Leukotriene B4
LA	Linoleic acid
LOX	Lipoxygenase
lyso-PAF	Lyso-Platelet-Activating Factor (1-O-hexadecyl-sn-glyceryl-3-phosphorylcholine)
MGD	Meibomian gland dysfunction
NSAID	Nonsteroidal anti-inflammatory drug
OEA	Oleoylethanolamine ((Z)-N-(2-Hydroxyethyl)octadec-9-enamide)
PBS	Phosphate-buffered saline
PUFA	Polyunsaturated fatty acid
PGD2	Prostaglandin D2
PGE2	Prostaglandin E2
PGF2α	Prostaglandin F2 alpha
ROS	Reactive oxygen species
TF	Tear fluid
TNF-α	Tumor necrosis factor alpha
UPLC-MS/MS	Ultra performance liquid chromatography-tandem mass spectrometry
